# Pachyonychie congénitale associée à une sténose de l’artère rénale et une dilatation des bronches

**DOI:** 10.11604/pamj.2016.24.183.9284

**Published:** 2016-07-01

**Authors:** El Alaoui Ismaili Fatiha, Chemlal Abdeljalil, Karimi Ilham, Benabdellah Nawal, Bentata Yassamine, Zizi Nada, Benajiba Nafissa, Haddiya Intissar

**Affiliations:** 1 Service de Néphrologie, CHU Mohamed VI, Faculté de Médecine, Université Mohamed Premier, Oujda, Maroc; 2 Service de Dermatologie, CHU Mohamed VI, Faculté de Médecine, Université Mohamed Premier, Oujda, Maroc; 3 Service de Néphrologie, CHU Mohamed VI, Faculté de Médecine, Université Mohamed Premier, Oujda, Maroc

**Keywords:** Pachyonychie congénitale, sténose de l´artère rénale, dilatation des bronches, Pachyonychia congenita, renal artery stenosis, bronchiectasis

## Abstract

La pachyonychie congénitale (PC) est une maladie héréditaire rare, essentiellement caractérisée par une kératodermie palmoplantaire douloureuse, un épaississement des ongles, des kystes et une muqueuse buccale blanchâtre. Sa présentation clinique est très variable, elle peut apparaître de la naissance à l'âge adulte. Cette observation rapporte l'association d'une pachyonychie congénitale avec une dilatation des bronches (DDB) et une sténose de l'artère rénale chez une enfant. le diagnostic de la pachyonychie était retenu sur les données cliniques et histologiques. Cependant l'existence d'une sténose de l'artère rénale et d'une DDB soulève la question d'une éventuelle association dans le cadre d'un groupe syndromique particulier.

## Abstract (en)

Pachyonychia congenita (PC) is a rare hereditary disease, mainly characterized by a painful palmoplantar keratoderma, thickened nails, cysts and white lesions of the oral mucosa. Its clinical manifestations are very variable, it may appear from birth to adulthood. This study report the case of a child with pachyonychia congenita associated with bronchiectasis and renal artery stenosis. The diagnosis of pachyonychia congenita was retained based on clinical and histological data. However the presence of renal artery stenosis and bronchiectasis suggests a possible association as part of a particular syndromic group.

## Introduction

La pachyonychie congénitale (PC) est une maladie héréditaire autosomique dominante, caractérisée majoritairement par une onychodystrophie hypertrophique et une kératodermie palmo-plantaire chez plus de 90% des patients [1]. Cette pathologie est causée par l'une des mutations des gènes de la kératine, et était historiquement divisée en deux sous-groupes, la PC1et la PC2, selon la présentation clinique. Actuellement des nomenclatures plus spécifiques basées sur la mutation génétiques causales sont adoptées [2]. Cependant, des formes récessives et des formes sporadiques sont également rapportées [3]. Cette maladie se rencontre chez le nouveau-né et l´enfant en bas âge, et se caractérise par une hyperkératose, c´est-à-dire une augmentation de l'épaisseur de la couche cellulaire constituant l´épiderme et contenant de la kératine. EIle peut s'associer à une surdité, une cataracte, une dyskératose cornéenne, une leucokératose de la langue, des formations kystiques, une hyperhidrose palmo-plantaire ainsi que des folliculites kératosiques au niveau du tronc et des extrémités. La présence permanente de ces ongles sous forme de cornes conditionne la qualité de vie des patients et impose dans certains cas une excision de la matrice afin de freiner leur croissance [4, 5]. Nous rapportons à travers cette observation, un cas sporadique d'une pachyonychie congénitale associée à une sténose idiopathique de l'artère rénale et une dilatation des bronches (DDB) chez une enfant.

## Patient et observation

**Figure 1:**
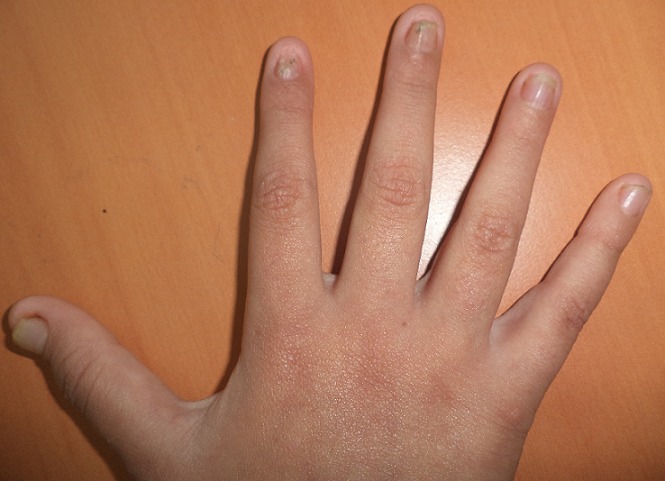
Pachyonychie des doigts chez notre patiente

**Figure 2:**
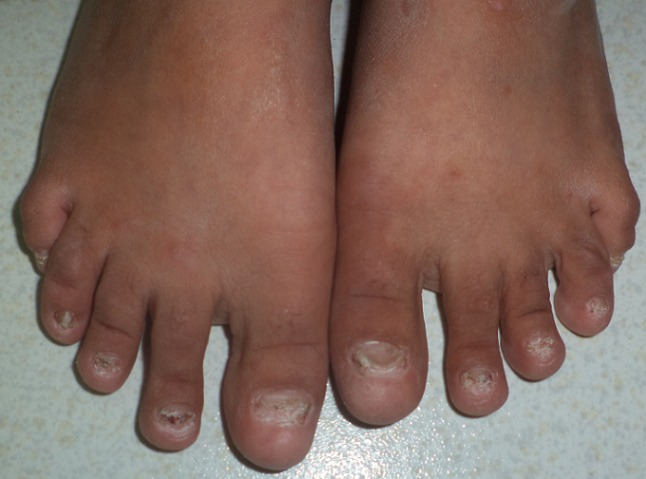
Pachyonychie des orteils chez notre patiente

**Figure 3:**
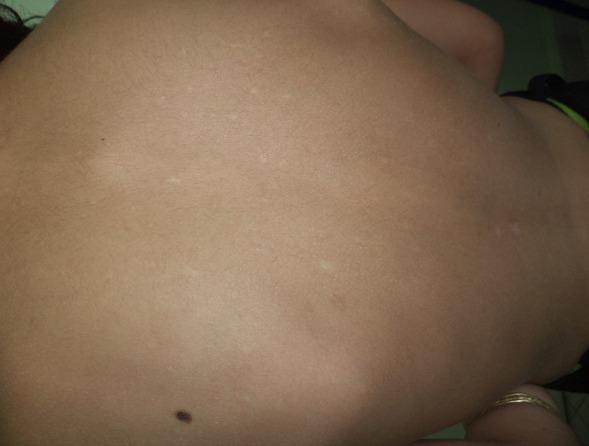
Kératose du tronc chez notre patiente

**Figure 4:**
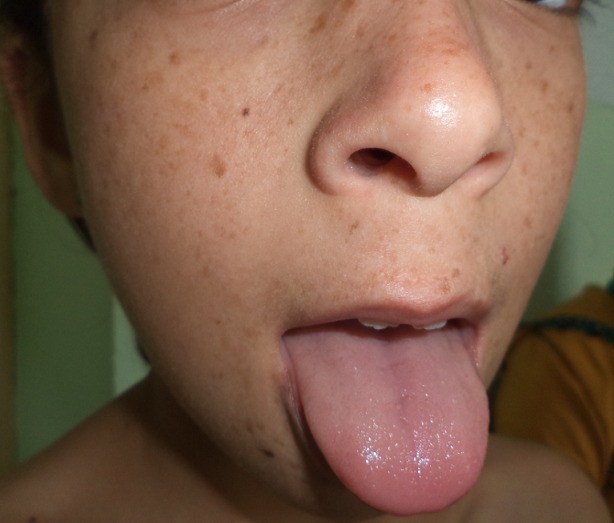
Leucokératose de la langue chez notre patiente

Il s'agit d'une enfant caucasienne, âgée de neuf ans, issue d'un mariage consanguin, et deuxième d'une fratrie de trois, qui présente une pachyonychie des ongles des dix doigts et des dix orteils, une kératose douloureuse des membres, et une kératose du tronc, découverte pendant la première année de vie (Figure 1, Figure 2, Figure 3, Figure 4). Dans ses antécédents, on note des broncho-alvéolites à répétition dès l'âge de sept mois, et une invagination intestinale à l'âge de dix-huit mois traitée par une insufflation. A l'âge de huit ans, elle avait présenté une insuffisance cardiaque globale avec une hypertension artérielle sévère. Le bilan cardiaque avait objectivé une cardiomyopathie dilatée hypertensive, et le bilan étiologique de l'hypertension artérielle (HTA) avait révélé une sténose de l'artère rénale gauche au niveau ostial avec une absence totale de parenchyme rénal gauche fonctionnel, Justifiant une néphrectomie. Aucune cause de la sténose de l'artère rénale n'était retrouvée chez notre patiente. Trois années plus tard, la patiente avait présenté une hypertrophie compensatrice du rein controlatéral avec une protéinurie néphrotique dans un contexte d'HTA. L'enfant était mise sous inhibiteurs de l'enzyme de conversion (IEC) à la dose quotidienne de cinq milligrammes, associés à un inhibiteur calcique à dix milligrammes par jour, avec un bon équilibre des chiffres tensionnels et une diminution de la protéinurie. A l'admission dans notre structure, à l'âge de neuf ans, l'examen clinique de la patiente avait objectivé, outre l'HTA, un épaississement des ongles et des orteils, une leucokératose de la langue, une hyperhidrose palmo-plantaire ainsi que des folliculites kératosiques au niveau du tronc. L'ensemble de ces lésions serait apparu à l'âge de trois mois selon la mère de l'enfant. Cependant, la patiente n'était pas suivie pour ces anomalies. Par ailleurs, l'examen n'avait pas montré de formations kystiques, ni de lésions évidentes au niveau capillaire, oculaire ou dentaire. De plus, les examens spécialisés ORL et ophtalmologique étaient sans particularités. L'enfant avait également bénéficié d'un examen mycologique, qui était négatif, ainsi que d'une biopsie unguéale dont le résultat était en faveur d'un épaississement unguéal avec une hyperplasie malpighienne concordant avec le diagnostic d'une pachyonychie congénitale. Compte tenu de ces différentes données et notamment de la présentation clinique, Il s'agit probablement d'une pachyonychie congénitale type I. En l'absence d'un traitement curatif, L'enfant avait reçu une médication à usage local à base de dérivés de la vitamine A, à savoir l'Acitrétine, à la dose de 10 mg/j pendant un mois. La patiente avait présenté une bonne tolérance du traitement et une amélioration de l'hyperkératose palmo-plantaire. Cependant, elle n'avait pas bénéficié de l'exérèse de la matrice unguéale, étant donné que la longueur et l'aspect des ongles, ne justifiaient pas ce geste. En outre, l'enfant continuait à présenter des infections bronchiques à répétition, sans organomégalie ni polyadénopathie, ni retard staturo-pondéral. Un déficit immunitaire était écarté devant la normalité de la numération sanguine et le dosage pondéral des immunoglobulines. Un scanner thoraco-abdomino-pelvien, en revanche, avait montré une dilatation des bronches localisée au niveau des lobes inférieurs bilatéraux, sans autres anomalies associées. La patiente est traitée régulièrement par des séances de kinésithérapie respiratoire et une antibiothérapie lors des épisodes de surinfection bactérienne.

## Discussion

Nous rapportons une association exceptionnelle entre une pachyonychie congénitale de type I, une dilatation des bronches et une sténose de l'artère rénale chez une enfant de neuf ans, issue d'un mariage consanguin, mais sans autres cas similaires dans la famille. La pachyonychie congénitale (PC) est une génodermatose rare, principalement caractérisée par une kératodermie palmoplantaire douloureuse, un épaississement des ongles, des kystes et une muqueuse buccale blanchâtre. Sa prévalence est inconnue, mais près de 1 000 cas ont été recensés dans le monde [3–7]. Au cours de la pachyonychie congénitale, on observe une transformation de la kératine et des couches superficielles de la peau (épiderme) ou de certaines muqueuses (couches cellulaires recouvrant l´intérieur des organes creux en contact avec l´air) qui s´enrichissent progressivement de kératine [3–5]. Cette kératose est située sous les ongles, sur la plante des pieds et sur la paume des mains. Elle apparaît sous la forme d' îlots associés à une hyperhidrose. Il existe, par ailleurs, une kératose pilaire avec un état de sécheresse de la peau et de petites élévations de la couche cornée, sèches, avec des plaques blanches au niveau de la langue [8, 9].

Dans le cas que nous rapportons, notre jeune patiente présentait l'ensemble de ces lésions. Deux types d'anomalies génétiques en terme de pachyonychie, la première correspond à des mutations qui affectent les kératines K6a, k6b et K16 et la seconde correspond à des modifications de la Kératine K17, exprimées dans des épithéliums divers [4, 5]. Par ailleurs, on décrit quatre types de pachyonychie congénitale. Le type I, correspondant au syndrome de Jadassohn-Lewandowski, associant une hypertrophie unguéale, une hyperkératose palmoplantaire, une kératose folliculaire et une leucokératose orale. Le type II ou syndrome de Jackson-Lawler, correspond au type I avec, des bulles palmoplantaires, une hyperhidrose palmoplantaire, une éruption précoce des dents ou des dents néonatales et des stéatocystomes multiples qui débutent habituellement après la puberté. Le type III est caractérisé par les signes cliniques du type II auxquels s'associent une dyskératose cornéenne, une cataracte et une perlèche. Le type IV, rarement décrit, comprend les signes décrits dans les trois types précédents, avec en plus des lésions laryngées, une raucité de la voix, un retard mental [3–6]. Au vu de sa présentation clinique, notre patiente correspond à la description du type I de la maladie. Il n´existe actuellement pas de traitement curatif de la maladie.

La prise en charge des patients est essentiellement symptomatique reposant sur le meulage des ongles et le contrôle de la douleur due à la kératodermie palmoplantaire, avec des soins émollients pour réduire l´hyperkératose et des stratégies pour limiter les frictions et traumatismes du pied [10]. De nouvelles perspectives thérapeutiques sont en cours d'évaluation, telles que les stratégies par les petits ARN interférents, les inhibiteurs de la mTOR par voie locale et systémique et l´injection de toxine botulique [10]. Dans la présente observation, notre patiente était traitée par des dérivés de la vitamine A avec une bonne réponse et une tolérance optimale. Concernant la sténose de l'artère rénale, les atteintes rénovasculaires sont responsables de 5 à 10 % des cas d'HTA observés chez l'enfant, car contrairement à l'adulte les étiologies sont dominées par la dysplasie fibromusculaire. Il est à noter qu'un certain nombre de pathologies syndromiques telle, la neurofibromatose de type 1, la sclérose tubéreuse de Bourneville peuvent être associées à la sténose de l'artère rénale chez l'enfant. Les vascularites, notamment la maladie de Takayashu, la périartérite noueuse, le syndrome de Kawasaki; ainsi que les compressions extrinsèques peuvent également s'y associer [9]. Dans le cas que nous rapportons, il n'y avait pas d'arguments cliniques, biologiques ni morphologiques en faveur de ces pathologies. A notre connaissance, il s'agit du premier cas rapporté dans notre pays. La rareté de la pathologie décrite ainsi que la singularité de l'association soulève un certain nombre d'interrogations; s'agit-il d'une association fortuite ou d'un ensemble pouvant s'intégrer dans un groupe syndromique particulier, non encore défini?

## Conclusion

La découverte d'une pachyonychie chez un enfant exige une exploration approfondie à la recherche des anomalies associées évidentes ou occultes, qui pourraient orienter vers un regroupement syndromique particulier.
